# Hematological Indices as Potential Biomarkers of Disease Activity in Ankylosing Spondylitis: LASSO-Based Multivariable Modelling

**DOI:** 10.3390/medicina62030497

**Published:** 2026-03-06

**Authors:** Sema Kaymaz-Tahra, Cansın Taşkın, Alpaslan Tanoglu

**Affiliations:** 1 Division of Rheumatology, Department of Internal Medicine, Faculty of Medicine, Bahcesehir University, Istanbul 34732, Turkey; 2 Department of Internal Medicine, Faculty of Medicine, Bahcesehir University, Istanbul 34732, Turkey; cansin.taskin@bau.edu.tr; 3 Division of Gastroenterology, Department of Internal Medicine, Faculty of Medicine, Bahcesehir University, Istanbul 34732, Turkey; alpaslantanoglu@yahoo.com

**Keywords:** ankylosing spondylitis, disease activity, hematological indices

## Abstract

*Background and Objectives*: Reliable laboratory markers that accurately reflect disease activity in ankylosing spondylitis (AS) are limited. Conventional acute-phase reactants do not consistently correlate with clinical activity. Composite hematological indices derived from complete blood count may better capture systemic inflammatory burden. In this study, we aimed to investigate hematologic parameters in AS and to assess their relationships with disease activity. *Materials and Methods*: This retrospective observational study included 196 patients with AS. Disease activity was defined as a Bath Ankylosing Spondylitis Disease Activity Index (BASDAI) ≥4. Demographic variables, laboratory parameters, hematological indices, and extra-articular manifestations were evaluated. Variable selection was performed using least absolute shrinkage and selection operator (LASSO) regression with ten-fold cross-validation. Variables with non-zero coefficients were entered into a multivariable logistic regression model. Model performance was assessed using receiver operating characteristic (ROC) curve analysis. *Results*: Ninety-seven (49%) patients had active disease. LASSO regression identified erythrocyte sedimentation rate (ESR), white blood cell count, red cell distribution width (RDW), platelet-to-lymphocyte ratio (PLR), and selected extra-articular manifestations as relevant predictors. In multivariable logistic regression, ESR (OR 1.03, 95% CI 1.00–1.06), white blood cell count (OR 1.23, 95% CI 1.04–1.46), and PLR (OR 1.01, 95% CI 1.003–1.020) were independently associated with active disease, while RDW showed a borderline association. The model demonstrated good discriminative ability (AUC 0.77, 95% CI 0.69–0.84). *Conclusions*: PLR is independently associated with disease activity in ankylosing spondylitis and improves discrimination when incorporated into a multivariable model. Easily accessible hematological indices may complement traditional inflammatory markers in the assessment of disease activity in routine clinical practice.

## 1. Introduction

Ankylosing spondylitis (AS) is a chronic inflammatory rheumatic disease that primarily affects the sacroiliac joints and axial skeleton, leading to pain, stiffness, and progressive structural damage [1,2]. The assessment of disease activity in AS is mainly based on clinical indices such as the Bath Ankylosing Spondylitis Disease Activity Index (BASDAI) and the Ankylosing Spondylitis Disease Activity Score (ASDAS), together with laboratory markers such as erythrocyte sedimentation rate (ESR) and C-reactive protein (CRP). However, these acute-phase reactants (APR) do not always correlate with clinical disease activity, as a considerable proportion of patients with active disease may present with normal CRP or ESR values [3].

In recent years, several hematological indices derived from routine complete blood count (CBC)—such as the neutrophil-to-lymphocyte ratio (NLR), platelet-to-lymphocyte ratio (PLR), mean platelet volume (MPV), and red cell distribution width (RDW)—have emerged as potential markers of systemic inflammation [4,5,6]. These parameters reflect the dynamic balance between inflammation and immune regulation. Zeb et al. demonstrated that both NLR and PLR were significantly elevated in AS patients compared with healthy controls and were positively correlated with disease activity, suggesting their potential as accessible inflammatory indicators [7]. Similarly, Liang et al. identified PLR as an independent factor associated with AS severity and found that higher PLR levels were observed in active AS patients [6].

Platelet indices such as MPV also provide insight into platelet activation and systemic inflammation [8]. Kisacik et al. reported that MPV values were significantly lower in patients with active AS and rheumatoid arthritis than in healthy subjects and increased significantly following anti-inflammatory treatment, indicating an inverse relationship between MPV and inflammatory activity [4]. A recent meta-analysis by Song and Lee demonstrated that RDW was significantly higher in AS patients compared with controls, and RDW, PLR, and MPV were all positively correlated with CRP levels, highlighting their potential utility as inflammatory biomarkers [5].

Reliable biomarkers reflecting disease activity in AS remain limited and composite hematological indices may capture inflammatory burden beyond conventional APR. The present study aimed to assess these hematologic parameters in AS patients and to investigate their relationships with disease activity.

## 2. Materials and Methods

### 2.1. Study Design and Setting

This retrospective observational study was conducted among consecutive patients admitted to the Rheumatology outpatient clinic of Bahcesehir University Medicalpark Hospital Turkiye, between November 2023 and September 2025. Data collection was carried out via systematic review of electronic hospital records over a 3-month period.

### 2.2. Study Population

Patients aged ≥18 years who were diagnosed with ankylosing spondylitis were included in the study. All the patients met either the 1984 modified New York Criteria for AS or the Assessment of Spondyloarthritis international Society (ASAS) classification criteria for axial Spondyloarthritis (axSpA) [9,10]. Exclusion criteria were patients under 18 years of age, patients lacking a CBC, patients with any kind of infection, individuals with cirrhosis, renal failure and patients who had a history of malignancy.

### 2.3. Data Collection

Demographic, clinical data and laboratory parameters were extracted from hospital records. Disease activity was assessed using BASDAI. Disease activity was primarily defined as BASDAI ≥ 4. For sensitivity analyses, active disease was alternatively defined as ASDAS-CRP ≥ 2.1.

Complete blood count-derived inflammatory ratios were calculated using absolute cell counts obtained from routine peripheral blood analysis at the time of clinical assessment. Platelet-to-lymphocyte ratio (PLR) was calculated as the absolute platelet count divided by the absolute lymphocyte count, platelet-to-monocyte-ratio (PMR) was found as the absolute platelet count divided by the absolute monocyte count, monocyte-to-lymphocyte-ratio (MLR) calculated as the absolute monocyte count divided by the absolute lymphocyte count, monocyte-to-neutrophil ratio (MNR) was calculated as the absolute monocyte count divided by the absolute neutrophil count, platelet-to-neutrophil ratio (PNR) was calculated as the absolute platelet count divided by the absolute neutrophil count. All ratios were derived from the same blood sample and analyzed as continuous variables without prior transformation.

Radiographic sacroiliitis was assessed on conventional pelvic radiographs according to the New York criteria, in which sacroiliac joints are graded on a scale from 0 to 4: grade 0, normal; grade 1, suspicious changes; grade 2, minimal abnormalities with small localized areas of sclerosis or erosions; grade 3, definite abnormalities including marked sclerosis, erosions, joint space narrowing or widening, or partial ankylosis; and grade 4, complete ankylosis of the sacroiliac joint. For classification purposes, radiographic sacroiliitis was considered present when at least bilateral grade ≥ 2 or unilateral grade ≥ 3 sacroiliitis was observed, in accordance with the modified New York criteria [9]. For each patient, the maximum sacroiliitis grade was defined as the higher grade observed between the right and left sacroiliac joints, and this value was used for subsequent analyses.

Active sacroiliitis on magnetic resonance imaging (MRI) was defined according to ASAS criteria as the presence of bone marrow oedema highly suggestive of inflammation, located in typical subchondral or periarticular regions of the sacroiliac joints. This was considered present if bone marrow oedema was observed on at least one slice in both sacroiliac joints (bilateral single-slice involvement) or on two or more consecutive slices in a single sacroiliac joint (unilateral multi-slice involvement) [11].

This current study was conducted in accordance with the Declaration of Helsinki and was approved by Sancaktepe Training and Research Hospital ethical committee (initial approval date: 17 March 2022). Considering the time elapsed since the initial approval, a renewed approval was obtained from the same Institutional Review Board on 29 August 2025 (No: E-46059653-020; E-46059653-050.04-286683424 (renewed)).Written informed consent was obtained from all patients prior to inclusion.

### 2.4. Statistical Analysis

The statistical analysis was performed using Statistical Package for the Social Sciences 22.0 (SPSS, Chicago, IL, USA) and R software version 4.5.2 (R Foundation for Statistical Computing, Vienna, Austria). The frequencies of the categorical data were expressed as number and percentages. The continuous variables were shown as median (min-max) or mean ± SD according to distribution. Chi square or Fisher exact test were used to compare the categorical variables and the continuous data was analyzed with Mann–Whitney U test.

Candidate demographic, laboratory, haematological, and extra-articular variables were evaluated to identify risk factors associated with active disease (BASDAI ≥ 4). The full set of candidate predictors included demographic variables (age, sex), disease-related characteristics (disease duration, HLA-B27 status), laboratory parameters (ESR, CRP, WBC, hemoglobin, platelet count, MCV, RDW, MPV), complete blood count-derived inflammatory ratios (PLR, PMR, MLR, MNR, NLR, PNR), extra-articular manifestations (uveitis, psoriasis, inflammatory bowel disease, enthesitis), imaging findings (presence of maximum radiographic sacroiliitis grade 3 or 4, MRI-defined active sacroiliitis), and treatment-related variables (current NSAID and biologic use). Least absolute shrinkage and selection operator (LASSO) regression with cross-validation was applied for variable selection. Variables with non-zero coefficients were subsequently entered into a multivariable logistic regression model. To enhance transparency and reproducibility, the full specification of the final multivariable logistic regression model, including regression coefficients (β), standard errors, odds ratios, and the model intercept, is provided in Appendix A Table A1. Model performance was assessed using receiver operating characteristic (ROC) analysis. A two-sided *p* value < 0.05 was considered statistically significant. Missing data were minimal and analyses were performed using a complete-case approach.

As a sensitivity analysis, disease activity was additionally assessed according to ASDAS-CRP, with scores ≥ 2.1 classified as active disease; parameters associated with active disease were re-selected using LASSO regression, and the logistic regression analysis was subsequently repeated based on this definition. For the ASDAS-CRP-based sensitivity analysis, odds ratios were not reported due to quasi-complete separation and instability of coefficient estimates; therefore, model performance was assessed using ROC analysis.

## 3. Results

A total of 196 (Female/Male: 96/100) AS patients were involved in the study. Mean age was 41.2 ± 12.9 years. Thirty-three (17%) patients had uveitis, 10 (5%) patients had psoriasis and 17 (9%) of the patients had concomitant inflammatory bowel disease (IBD). Mean disease duration was 5.8 ± 5.6 years. HLA-B27 was positive in 58% (72/125) patients.

Mean BASDAI score was 3.5 ± 2.4. Ninety-seven (49%) patients had active disease (BASDAI ≥ 4). The proportion of male patients was lower among those with active disease (Active vs. inactive proportion of male patients 43% vs. 59%, *p* = 0.032). HLA-B27 positivity (Active vs. inactive 59% vs. 58%, *p* = 0.84) and disease duration (Active vs. inactive: 6.7 ± 7.3 vs. 4.9 ± 3.2 years, *p* = 0.82) were similar between groups. APRs were significantly higher in patients with active disease (ESR active vs. inactive 24.2 ± 19.5 vs. 14.5 ± 10.1 mm/h, *p* = 0.001) (CRP active vs. inactive 3.7 (0.5–12.1) vs. 2.9 (0.2–26.2) mg/L, *p* = 0.004). The hemoglobin (13.9 ± 1.6 vs. 14.4 ± 1.8 g/dL, *p* < 0.01) and MCV (85.3 ± 4.5 vs. 87.0 ± 3.7 fL, *p* = 0.018) levels were significantly lower in the active group. RDW (13.7 ± 1.1 vs. 13.0 ± 0.7, *p* < 0.01) and PLR (153.7 ± 72.1 vs. 111.6 ± 42.5, *p* = 0.002) were higher in active AS patients compared to inactives (Table 1). MNR was significantly lower and NLR was higher in active group while PMR, MLR and PNR were similar between groups (Table 1).

Active sacroiliitis on MRI was observed in 74% (100/135) of the patients. Conventional pelvic radiographs were available for 159 patients. Based on the maximum sacroiliitis grade, 9 patients (5.7%) had grade 1, 51 patients (32.1%) had grade 2, 81 patients (50.9%) had grade 3, and 18 patients (11.3%) had grade 4 sacroiliitis.

The frequency of maximum sacroiliitis grade 3 or 4 in Xray and active sacroiliitis on MRI were similar in patients who had active disease and who had inactive disease according to BASDAI (Table 1).

Current treatments included nonsteroidal anti-inflammatory drugs (NSAIDs) in 119 patients (61%), tumor necrosis factor inhibitors in 66 patients (33%), secukinumab in 9 patients (5%), and upadacitinib in 1 patient. NSAID use was more frequent in the active disease group (70% vs. 52%, *p* = 0.008) whereas the use of biologics was less frequent among patients with active disease (30% vs. 47%, *p* = 0017) (Table 1).

### 3.1. Variable Selection by LASSO Regression

**Figure 1:**
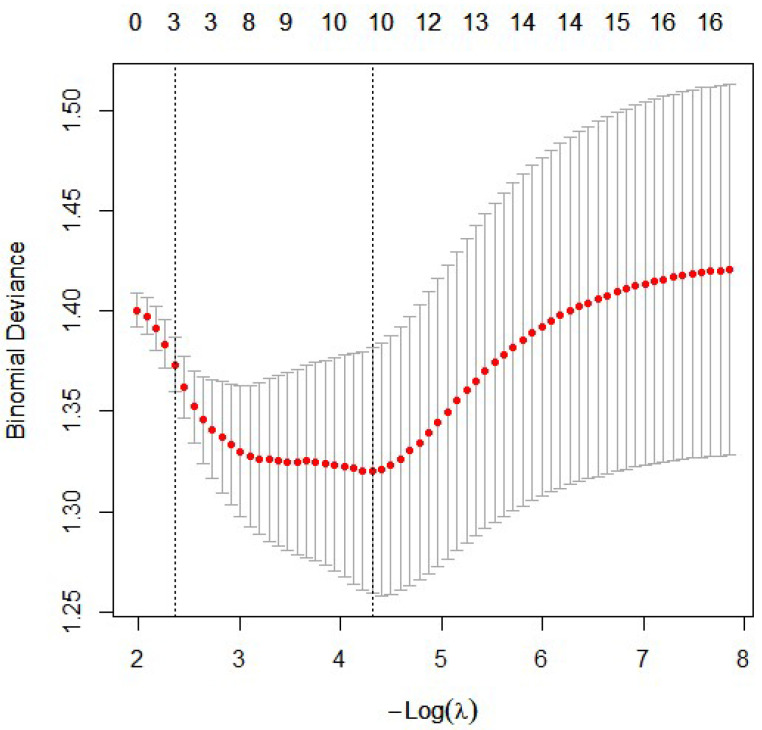
Least absolute shrinkage and selection operator (LASSO) regression was applied to identify predictors of active disease (BASDAI ≥ 4). The figure shows the cross-validated binomial deviance plotted against log(λ). The red dots represent the mean cross-validated error, and the vertical bars indicate ±1 standard error. The left vertical dashed line corresponds to the optimal penalty parameter (λ_min), yielding the minimum cross-validated deviance, while the right dashed line indicates the largest penalty within one standard error (λ_1se). Numbers along the top axis represent the number of variables retained at each λ. At λ_min, a total of nine variables with non-zero coefficients were selected for subsequent multivariable logistic regression analysis.

Using LASSO regression, nine variables with non-zero β coefficients were selected as potential predictors of active disease. These included ESR (β = 0.024), white blood cell (WBC) count (β = 0.175), RDW (β = 0.223), PLR (β = 0.010), female sex (β = 0.351), uveitis (β = −0.414), psoriasis (β = 0.985), inflammatory bowel disease (IBD) (β = 0.335), and enthesitis (β = 0.243). Age, CRP, platelet count, MCV, and MPV were excluded from the final model (Figure 1).

### 3.2. Multivariable Logistic Regression

In the multivariable logistic regression model, ESR (OR 1.03, 95% CI 1.00–1.06, *p* = 0.039), WBC (OR 1.23, 95% CI 1.04–1.46, *p* = 0.016), and PLR (OR 1.01, 95% CI 1.00–1.02, *p* = 0.013) were independently associated with active disease (BASDAI ≥ 4). RDW showed a borderline association with disease activity (OR 1.31, 95% CI 1.03–1.78, *p* = 0.051) (Table 2). The complete model specification, including regression coefficients and the intercept, is presented in Appendix A Table A1.

### 3.3. Model Performance

**Figure 2:**
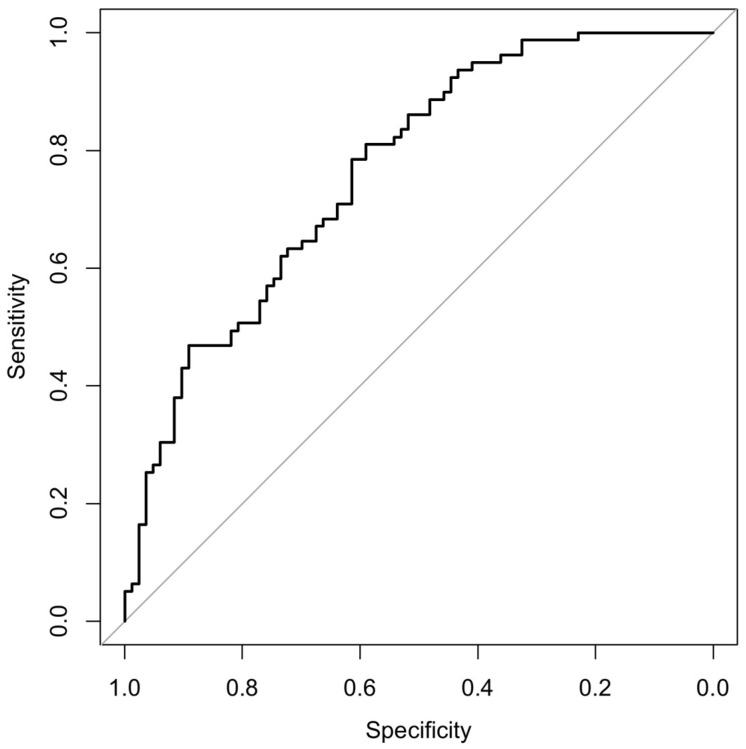
Receiver operating characteristic (ROC) curve for active disease. Receiver operating characteristic (ROC) curve of the multivariable logistic regression model predicting active disease (BASDAI ≥ 4). The model demonstrated good discriminative performance with an area under the curve (AUC) of 0.77 (95% CI 0.69–0.84). Variables included in the model were selected using least absolute shrinkage and selection operator (LASSO) regression.

ROC analysis demonstrated good discriminative performance of the final model, with an area under curve (AUC) of 0.77 (95% CI 0.69–0.84), indicating acceptable ability to distinguish active disease from inactive disease (Figure 2).

Using ROC curve analysis, the optimal model-based probability cut-off determined by the Youden index was 0.396, yielding a sensitivity of 81.0% and a specificity of 59.0% (AUC 0.77, 95% CI 0.69–0.84). In contrast, PLR alone showed moderate discriminative performance, with an AUC of 0.63 (95% CI 0.55–0.72); the optimal PLR cut-off was 133.1, providing a sensitivity of 49.4% and a specificity of 74.7%.

**Figure A1:**
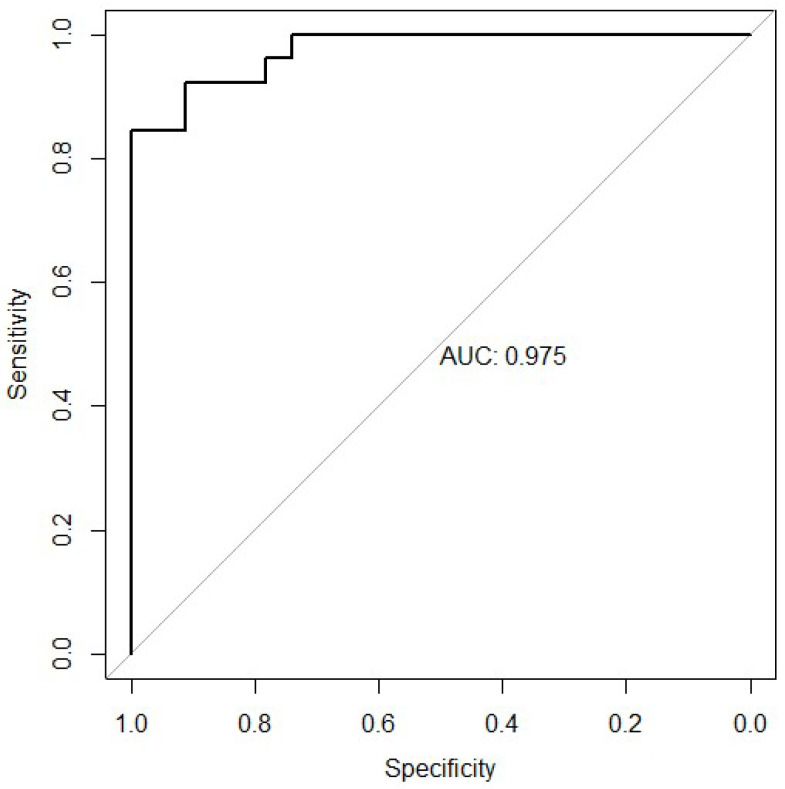
Receiver operating characteristic (ROC) curve showing the discriminative performance of the expanded multivariable logistic regression model including all complete blood count-derived haematological ratios (PMR, MLR, MNR, NLR, PNR) and clinical covariates for identifying active disease (BASDAI ≥ 4). The model demonstrated excellent discrimination, with an area under the curve (AUC) of 0.975. The diagonal line represents the line of no discrimination.

In the expanded multivariable logistic regression model including all complete blood count-derived haematological ratios (PMR, MLR, MNR, NLR, PNR) and clinical covariates, none of the individual predictors demonstrated an independent association with BASDAI-defined active disease when assessed according to the 95% confidence interval criterion, as all confidence intervals crossed unity. RDW showed a statistically significant *p* value; however, the corresponding confidence interval was wide, indicating substantial uncertainty in the effect estimate. Extra-articular manifestations and treatment-related variables were also associated with wide confidence intervals, likely reflecting shared biological information and collinearity among these indices (Appendix A Table A2). Despite the lack of statistically significant individual predictors, the combined model demonstrated excellent discriminative performance, with an area under the curve (AUC) of 0.97 (95% CI 0.94–1.00), indicating strong overall ability to distinguish between patients with active and inactive disease (Appendix A Figure A1).

**Figure A2:**
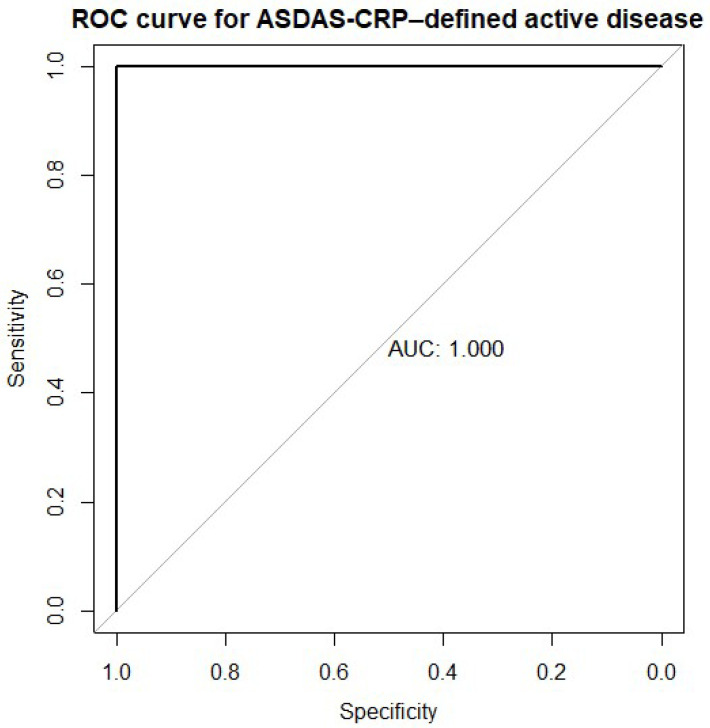
Receiver operating characteristic (ROC) curve illustrating the discriminative performance of the multivariable model for identifying active disease defined according to ASDAS-CRP criteria. The model demonstrated excellent discrimination, with an area under the curve (AUC) of 1.00.

Using ASDAS-CRP-defined activity as a sensitivity analysis, LASSO regression selected a similar but not identical set of predictors. The resulting model demonstrated excellent discriminative performance (AUC 0.97) (Appendix A Figure A2). However, due to quasi-complete separation and extreme coefficient estimates, individual odds ratios were not interpretable and therefore not reported.

## 4. Discussion

In this study, we identified a multivariable model incorporating inflammatory and haematological parameters that was independently associated with disease activity in AS and demonstrated good discriminative performance. Among the selected variables, ESR, WBC, RDW, and PLR were independently associated with active disease. Notably, PLR remained significantly associated with disease activity even after adjustment for conventional inflammatory markers and extra-articular manifestations. Although several other complete blood count-derived indices were also examined, their associations with disease activity were less consistent after multivariable adjustment, highlighting the relative stability of PLR among the evaluated haematological markers.

Although acute-phase reactants such as ESR and CRP are frequently used in the assessment of disease activity in AS, their levels may not always correlate with clinical findings. Moreover, inflammatory cytokines such as interleukin-6 (IL-6) and tumor necrosis factor-alpha (TNF-α) have been shown to reflect disease activity in AS, but are not routinely assessed in clinical practice [12,13,14]. Previous studies have shown only modest correlations between ESR and CRP with clinical activity indices such as BASDAI, and many patients with clinically active disease do not demonstrate elevated ESR or CRP levels highlighting a significant discordance between laboratory inflammation and patient-reported activity scores such as BASDAI [3,15]. Consistent with this heterogeneity, ESR remained retained in the final LASSO-selected multivariable model, whereas CRP was not, suggesting differential contributions of these acute-phase reactants to the assessment of disease activity in our cohort.

Several haematological indices have been investigated as potential markers for assessing disease activity in AS and axSpA in recent studies. These studies have examined indices such as PLR, NLR, monocyte-related indices, and composite inflammatory markers in relation to clinical disease activity and inflammatory burden. Previous studies have indicated that hematological indices derived from the complete blood count could complement conventional inflammatory markers in multivariable models of disease activity and severity [6,16,17]. In addition, treatment-oriented cohorts have explored longitudinal changes in these indices following biologic therapy, suggesting potential responsiveness to inflammatory control, although results remain heterogeneous across indices and outcome measures [18,19]. Notably, a very recent study further expanded this field by systematically evaluating multiple hematological indices in relation to disease activity in axial spondyloarthritis, reinforcing the concept that CBC-derived markers may reflect distinct aspects of systemic inflammation beyond traditional acute-phase reactants [20].

One of these hematological indices, PLR, may reflect complementary inflammatory processes involving platelet activation and relative lymphopenia. Platelets are increasingly recognized as active participants in inflammatory and immune pathways through the release of cytokines/chemokines and through interactions with leukocytes and endothelial cells [21,22,23]. In a large cohort study, Liang et al. demonstrated that PLR was not only elevated in AS patients compared with controls but also positively correlated with disease severity, BASDAI scores, and radiographic sacroiliitis grades. Importantly, PLR was identified as an independent factor associated with AS severity [6]. In line with these findings, PLR in our cohort was independently associated with clinical disease activity, even after adjustment for conventional inflammatory markers and extra-articular manifestations. Similarly, Zeb et al. reported significantly higher PLR values in patients with active AS compared with those in remission, while ESR failed to reliably distinguish disease activity states [7]. Our findings corroborate this evidence and further show that PLR remains independently associated with disease activity even after adjustment for traditional inflammatory markers. While PLR alone showed moderate discriminative performance, with higher specificity than sensitivity, overall discrimination was substantially improved in the multivariable model incorporating PLR alongside additional inflammatory and clinical variables. These findings support the role of PLR as a complementary biomarker rather than a standalone indicator of disease activity. When additional complete blood count-derived ratios were incorporated into the model, the independent statistical significance of individual haematological parameters diminished despite excellent overall discriminative performance, likely reflecting overlapping inflammatory information captured by these composite indices rather than a lack of biological relevance. As PLR integrates platelet activation and relative lymphopenia, it may serve as a representative surrogate of systemic inflammation. To preserve clinical interpretability and avoid redundancy, we therefore prioritized the PLR-based model as the primary analysis and presented the expanded haematological model as a secondary, exploratory analysis, with cut-off values interpreted cautiously in the absence of external validation.

RDW showed a borderline association with disease activity in our multivariable model. RDW has been proposed as a marker of chronic inflammation, potentially reflecting cytokine-mediated impairment of erythropoiesis and altered red blood cell maturation and has been shown to increase in chronic inflammatory states due to cytokine-mediated alterations in erythropoiesis [24,25]. A comprehensive meta-analysis demonstrated that RDW is consistently higher in patients with AS and is positively correlated with CRP levels, supporting its role as a marker of inflammatory burden [5]. Although RDW did not reach statistical significance in the final model, its selection during the LASSO step suggests that it may still provide complementary information regarding disease activity.

MPV, another platelet-related parameter, was not associated with disease activity in our cohort. Previous studies have yielded conflicting results regarding MPV in AS. Kisacik et al. reported significantly lower MPV values in patients with active AS compared with healthy controls, with MPV increasing after anti-inflammatory treatment, suggesting an inverse relationship between MPV and inflammation [4]. However, MPV is known to be relatively sensitive to pre-analytical factors, platelet turnover, and treatment-related changes, which may, at least in part, contribute to the lack of association observed in our study. Notably, a meta-analysis did not demonstrate a consistent difference in MPV between patients with AS and controls, despite reporting associations between MPV and inflammatory markers such as CRP [5].

WBC count independently predicted active disease in our analysis, reflecting generalized inflammatory activation. Although extra-articular manifestations such as uveitis, psoriasis, and inflammatory bowel disease were selected during the LASSO procedure, they did not retain significance in the fully adjusted model. Both clinical features and laboratory parameters in AS may be influenced by disease duration and treatment exposure. In this context, haematological indices may reflect certain aspects of concurrent systemic inflammation, whereas extra-articular manifestations often follow a heterogeneous and episodic course [26,27,28]. Accordingly, the lack of statistical significance of extra-articular manifestations in the fully adjusted multivariable model is most likely attributable to limited statistical power due to the relatively small number of events, rather than a true absence of association.

BASDAI is a purely patient-reported index that captures pain, stiffness, and fatigue—key symptomatic dimensions of disease activity that do not necessarily correlate with objective inflammatory markers. In contrast, ASDAS incorporates CRP as an integral component of the score. Given that the primary aim of this study was to evaluate the association between laboratory-derived inflammatory indices and clinical disease activity, using ASDAS-CRP as the primary outcome would have introduced mathematical coupling between predictors and outcome, potentially inflating associations and limiting interpretability. By focusing on BASDAI, we were therefore able to assess whether haematological parameters provide information beyond conventional acute-phase reactants.

When disease activity was alternatively defined using ASDAS-CRP in sensitivity analyses, the overall discriminative performance of the model increased substantially, likely reflecting the inclusion of objective inflammatory components within the outcome definition. However, this approach resulted in quasi-complete separation, which limited reliable estimation of individual effect sizes. These findings underscore the importance of parsimonious, clinically interpretable models—such as the PLR-based BASDAI analysis—for explanatory inference, while more complex ASDAS-based models may be more suitable for prediction-oriented sensitivity analyses. Despite these advances, data directly linking individual haematological indices to ASDAS-defined activity remain limited, highlighting the need for future prospective studies specifically addressing ASDAS-based outcomes.

By including patients who fulfilled either the modified New York criteria or the ASAS classification criteria, the study population encompassed the full spectrum of axSpA, including both radiographic and non-radiographic forms. Key disease-related characteristics, such as HLA-B27 status, radiographic sacroiliitis grading, and MRI-defined active sacroiliitis, were incorporated into the analyses. Disease duration and current treatments, including NSAIDs and biologic agents, were also considered, given their potential influence on both haematological indices and clinical disease activity. In our cohort, NSAID use was more frequent among patients with active disease, whereas biologic therapies were less commonly used in the active disease group, a distribution that likely reflects real-world treatment patterns in which NSAIDs are preferentially used during symptomatic flares and biologic therapies are prescribed to achieve and maintain disease control. Accordingly, treatment status should be interpreted as a marker of disease severity and management strategy rather than a causal determinant of disease activity. Although treatment exposure and disease chronicity may introduce heterogeneity in laboratory parameters and clinical manifestations, these factors were accounted for in the multivariable models. Nevertheless, the retrospective design limited precise evaluation of temporal relationships between disease onset, treatment initiation, and the evolution of extra-articular manifestations, underscoring the need for future prospective studies.

Several limitations of the present study should be acknowledged. This study has several limitations. Its single-center, retrospective design and the absence of an external validation cohort limit generalizability, and the findings should therefore be considered hypothesis-generating, although the use of penalized regression with cross-validation may have reduced the risk of overfitting. The inclusion of both radiographic and non-radiographic axial spondyloarthritis may have introduced clinical and biological heterogeneity, and imaging assessments at a single time point may not fully reflect dynamic inflammatory and structural changes. Disease activity was primarily defined using BASDAI, which is symptom-driven; although a sensitivity analysis using ASDAS-CRP was performed, the results may not fully capture inflammation-based disease activity. Although treatment status at the time of blood sampling was recorded and disease duration was included as a covariate, residual confounding related to treatment effects and long-term disease course cannot be excluded. Finally, detailed iron parameters were not routinely available, which may influence the interpretation of RDW-related findings, and the incremental predictive value of PLR should therefore be interpreted with caution.

## 5. Conclusions

In conclusion, the present study demonstrates that PLR is a clinically relevant, inexpensive, and easily obtainable marker associated with disease activity in AS. Beyond its individual association, PLR contributed meaningfully to multivariable models integrating inflammatory, clinical, and imaging-related parameters, thereby enhancing discrimination between active and inactive disease states. These findings support the complementary role of PLR alongside conventional inflammatory markers in clinical assessment. Nevertheless, further prospective studies with external validation are required to confirm the generalizability and clinical applicability of these results. Formal evaluation of clinical utility using decision-analytic methods was beyond the scope of the present study and would be required before integration into clinical decision-making frameworks.

## Figures and Tables

**Table 1:** Comparison of the characteristics of AS patients with active and inactive disease.

Variable	Total Group (*n* = 196)	Active Disease ^a^ (*n* = 97)	Inactive Disease ^a^ (*n* = 99)	*p* ^b^
Age, years	41.2 ± 12.9	33.8 ± 8.3	43.9 ± 7.3	0.75
Male gender, *n* (%)	100 (51)	42 (43)	58 (59)	0.032 *
HLA B27, positive, *n* (%)	72/125 (58)	38/65 (59)	34/60 (58)	0.84
Disease duration, years	5.8 ± 5.6	6.7 ± 7.3	4.9 ± 3.2	0.82
Uveitis, *n* (%)	33 (17)	13 (13)	20 (20)	0.20
Psoriasis, *n* (%)	10 (5)	6 (6)	4 (4)	0.49
IBD, *n* (%)	17 (9)	12 (12)	5 (5)	0.069
Peripheral arthritis, *n* (%)	37 (19)	21 (21)	16 (16)	0.32
Enthesitis, *n* (%)	32 (16)	18 (19)	14 (14)	0.40
BASDAI	3.5 ± 2.4	6.1 ± 0.8	1.8 ± 1.2	<0.01 *
Laboratory				
ESR, mm/h	11.9 ± 10.4	24.2 ± 19.5	14.5 ± 10.1	0.001 *
CRP, mg/L	3.2 (2–26)	3.7 (0.5–12.1)	2.9 (0.2–26.2)	0.004 *
WBC, 10^3^/uL	8.38 ± 1.79	8.8 ± 2.1	8.1 ± 1.6	0.20
Hgb, g/dL	14.2 ± 1.6	13.9 ± 1.6	14.4 ± 1.8	0.001 *
Plt, 10^3^/uL	282.8 ± 70.7	306.3 ± 31.7	267.9 ± 85.3	0.32
RDW, %	13.2 ± 0.9	13.7 ± 1.1	13.0 ± 0.7	<0.01 *
PLR	127.93 ± 57.83	153.7 ± 72.1	111.6 ± 42.5	0.002 *
PMR	527.0 ± 157.0	535.5 ± 170.9	521.5 ± 152.5	0.87
MLR	0.339 ± 0.381	0.364 ± 0.297	0.324 ± 0.435	0.11
MNR	0.128 ± 0.599	0.109 ± 0.054	0.141 ± 0.061	**0.048**
NLR	2.53 ± 2.07	3.78 ± 2.81	1.74 ± 0.76	**0.002**
PNR	66.46 ± 29.02	50.60 ± 16.44	76.72 ± 31.09	0.12
MPV, fL	9.9 ± 0.8	10.0 ± 1.2	9.9 ± 0.7	0.65
MCV, fL	86.9 ± 5.7	85.3 ± 4.5	87.0 ± 3.7	0.018 *
Xray, Max sacroiliitis grade 3 or 4	99/159 (62)	49/83 (59)	50/76 (66)	0.38
MRI, active sacroiliitis *	100/135 (74)	57/77 (74)	43/58 (74)	0.98
Treatment				
NSAIDs, *n* (%)	119 (61)	68 (70)	51 (52)	**0.008**
Biologic agents, *n* (%)	75 (38)	29 (30)	46 (47)	**0.017**
TNF inhibitors, *n* (%)	66 (34)	23 (24)	43 (43)	**0.003**
Secukinumab, *n* (%)	9 (5)	6 (6)	3 (3)	0.29

^a^ Patients who had ≥4 BASDAI were considered active and <4 was considered inactive according to BASDAI. ^b^ A *p* value < 0.05 was considered statistically significant. * Bold values indicate statistical significance. (*p* < 0.05) Laboratory parameters are presented using standardized units. Erythrocyte sedimentation rate (ESR) is expressed in mm/h (reference range: 0–20 mm/h), C-reactive protein (CRP) in mg/L (reference range: <5 mg/L), white blood cell count (WBC) in ×10^3^/µL (reference range: 4.0–10.0), hemoglobin (Hgb) in g/dL (reference range: 12–16 for females, 13–17 for males), platelet count (Plt) in ×10^3^/µL (reference range: 150–400), mean corpuscular volume (MCV) in fL (reference range: 80–100), red cell distribution width (RDW) in % (reference range: 11.5–14.5), mean platelet volume (MPV) in fL (reference range: 7.5–11.5). Reference ranges are provided according to institutional laboratory standards. The following hematological ratios were calculated from complete blood count parameters: platelet-to-monocyte ratio (PMR), calculated as platelet count divided by monocyte count; monocyte-to-lymphocyte ratio (MLR), calculated as monocyte count divided by lymphocyte count; monocyte-to-neutrophil ratio (MNR), calculated as monocyte count divided by neutrophil count; platelet-to-lymphocyte ratio (PLR), calculated as platelet count divided by lymphocyte count; neutrophil-to-lymphocyte ratio (NLR), calculated as neutrophil count divided by lymphocyte count; and platelet-to-neutrophil ratio (PNR), calculated as platelet count divided by neutrophil count.

**Table 2:** Independent predictors of active disease (BASDAI ≥ 4) identified by multivariable logistic regression after LASSO-based variable selection.

Variable	OR	95% CI	*p*
Gender, female	1.53	0.74–3.17	0.254
ESR, mm/h	1.03	1.00–1.06	**0.039**
WBC, 10^3^/µL	1.23	1.04–1.46	**0.016**
RDW, %	1.31	1.03–1.78	0.051
PLR	1.01	1.003–1.020	**0.013**
Uveitis	0.57	0.21–1.44	0.241
Psoriasis	4.09	0.66–34.37	0.146
IBD	1.86	0.46–8.29	0.387
Enthesitis	1.58	0.62–4.10	0.341

Values are presented as odds ratios (ORs) with 95% confidence intervals (CIs). Variables included in the multivariable model were selected using least absolute shrinkage and selection operator (LASSO) regression. A *p* value < 0.05 was considered statistically significant. Laboratory parameters are presented using standardized units. Erythrocyte sedimentation rate (ESR) is expressed in mm/h (reference range: 0–20 mm/h), white blood cell count (WBC) in ×10^3^/µL (reference range: 4.0–10.0), red cell distribution width (RDW) in % (reference range: 11.5–14.5), Reference ranges are provided according to institutional laboratory standards. The following hematological ratios were calculated from complete blood count parameters: platelet-to-monocyte ratio (PMR), calculated as platelet count divided by monocyte count; monocyte-to-lymphocyte ratio (MLR), calculated as monocyte count divided by lymphocyte count; monocyte-to-neutrophil ratio (MNR), calculated as monocyte count divided by neutrophil count; platelet-to-lymphocyte ratio (PLR), calculated as platelet count divided by lymphocyte count; neutrophil-to-lymphocyte ratio (NLR), calculated as neutrophil count divided by lymphocyte count; and platelet-to-neutrophil ratio (PNR), calculated as platelet count divided by neutrophil count.

## Data Availability

The data presented in this study are available on request from the corresponding author.

## Abbreviations

The following abbreviations are used in this manuscript:

AS	Ankylosing spondylitis
BASDAI	Bath Ankylosing Spondylitis Disease Activity Index
ASDAS	Ankylosing Spondylitis Disease Activity Score
ESR	Erythrocyte sedimentation rate
CRP	C-reactive protein
APR	Acute-phase reactants
CBC	Complete blood count
NLR	Neutrophil-to-lymphocyte ratio
PLR	Platelet-to-lymphocyte ratio
PMR	Platelet-to-monocyte ratio
MLR	Monocyte-to-lymphocyte ratio
MNR	Monocyte-to-neutrophil ratio
PNR	Platelet-neutrophil ratio
MRI	Magnetic resonance imaging
NSAIDs	Nonsteroidal anti-inflammatory drugs
MPV	Mean platelet volume
RDW	Red cell distribution width
LASSO	Least absolute shrinkage and selection operator
ROC	Receiver operating characteristic
IBD	Inflammatory bowel disease
WBC	White blood cell count
AUC	Area under the curve
IL-6	Interleukin-6
TNF-α	Tumor necrosis factor-alpha

## Appendix A

**Table A1:** Full specification of the final fitted multivariable logistic regression model for BASDAI-defined active disease (BASDAI ≥ 4).

Variable	β (Log-Odds)	SE	OR	95% CI (OR)	*p*
Intercept (β_0_)	−7.799	2.075	–	–	<0.001
Gender	0.393	0.370	1.48	0.72–3.06	0.289
ESR (sedimentation)	0.032	0.014	1.03	1.01–1.06	0.019
WBC	0.212	0.084	1.24	1.05–1.46	0.011
RDW	0.276	0.137	1.32	1.01–1.72	0.044
PLR	0.011	0.004	1.01	1.002–1.020	0.013
Uveitis	−0.579	0.487	0.56	0.22–1.46	0.235
Psoriasis	1.442	0.972	4.23	0.63–28.45	0.138
IBD	0.610	0.722	1.84	0.45–7.58	0.398
Enthesitis	0.459	0.481	1.58	0.62–4.06	0.340

Regression coefficients (β), standard errors (SE), odds ratios (OR), 95% confidence intervals (CI), and *p* values are presented. The model intercept is included to ensure full reproducibility.

**Table A2:** Multivariable logistic regression model including all complete blood count-derived haematological ratios and clinical variables for the prediction of BASDAI-defined active disease.

Variable	OR	95% CI	*p*
Disease duration	1.42	0.92–2.18	0.112
Female sex	6.11	0.13–288,876	0.191
ESR (sedimentation)	1.02	0.94–1.11	0.652
RDW	40.07	1.12–1428.31	0.043
Haemoglobin	2.98	0.42–20.91	0.272
MCV	0.91	0.56–1.50	0.719
Uveitis	1.78	0.03–99.75	0.779
Psoriasis	3.1 × 10^8^	0.00–∞	0.997
IBD	5.6 × 10^7^	0.00–∞	0.996
Enthesitis	0.01	0.00–2.94	0.112
Current NSAID use	1.80	0.11–2903.18	0.265
Current TNFi use	0.04	0.00–7.09	0.226

Odds ratios were derived from a multivariable logistic regression model including all haematological ratios selected by LASSO. Statistical significance was assessed based on the 95% confidence interval criterion; confidence intervals crossing unity were considered non-significant.
